# Sore, or Sour?

**Published:** 1869-05

**Authors:** 


### Editorial.

### SORE, OR SOUR?

We had a schoolmate once who never appeared to be happy, unless he had a sore toe. Little boys then and there ran barefoot. So by the aid of snags and sharp stones, an occasional cut with an axe, and especially assisted by the shoe-heels of the large boys, he could generally manage to be gratified; or what answered quite as well, he would imagine he had a sore toe and "tie it up," anyway. And how persistently he would patronize that sore toe! And how much deference he expected us all to show it! And how it was always a "very present help in time of trouble!" Behold, are not these all written in the book of the chronicles of the old brick schoolhouse?

That sore *toe* was a *handy* thing. If beaten at play, it was because he had a sore toe. If "turned down" in a spelling match, the boy that turned him down had hurt his sore toe. The teacher was expected to indulge him; for it would have been cruel to make him "toe the mark" with his sore toe. But, in spite of all his sufferings, he was a generous boy. If you would give him an apple he would show you his sore toe. We lost trace of our schoolmate; but we begin to suspect that he is now editing a professional journal. At any rate the "American Journal of Dental Science" has a sore toe, or thinks it has, which, as in the former case, answers just as well. And if the Dental profession in the South will give it an apple—no—an Association, it agrees to exhibit its sore toe for their peculiar gratification, and will not let the other boys see it at all.

The persistent effort of the "American Journal" to get up a prejudice in the South against the profession in the North is quite as silly, and far more wicked than the above. And it seems determined to succeed by means fair or foul. And we could not have been induced to allude to the controversy had it treated the DENTAL REGISTER's position with candor. The REGISTER published Dr. Morgan's reply to "J. H. McQ." in which Dr. M., alluding to the action of the Nominating Committee at the Chicago meeting, says, "And while the name of the writer was before the nominating Committee, he was approached by a member of that Committee direct from that Committee, and his politics asked. It was not doubted that the Committee sent him." Just at this point a foot note was added, in the REGISTER, by the Chairman of the nominating Committee, not only with Dr. Morgan's consent, but it was revised, as to language, by him. This note states positively, "He was not sent by the Committee." Now the *Journal*, in its zeal to "fire the southern heart," though taking the article from the REGISTER, fails to give the important fact contained in the foot note, which, be it remembered, is an *official* statement, and thus it does all it can to disgrace the nominating committee of the Chicago meeting, by allowing the silly twaddle and insulting conduct of a single member to be imputed to the whole Committeee, when it knew better. A cause that can only be carried by such means is worse than a sore toe.

We are in favor of a Southern Dental Association. In favor of a Northern one, a middle one—as many as there is room for, and we will attend as many as we can.

But, brethren, stop all this complaining about being slighted. The war was a great—a terrible fact; but it was fought through, and now has nothing to do with the progress of Dental science.

